# Sutureless Aortic Valve Replacement with Annular Plication Technique in an Oversized Annulus

**DOI:** 10.1093/icvts/ivaf287

**Published:** 2025-12-01

**Authors:** Shota Inoue, Chikara Ueki, Minoru Tabata

**Affiliations:** Department of Cardiovascular Surgery, Toranomon Hospital, Tokyo105-8470, Japan; Department of Cardiovascular Surgery, Toranomon Hospital, Tokyo105-8470, Japan; Department of Cardiovascular Surgery, Toranomon Hospital, Tokyo105-8470, Japan; Department of Cardiovascular Surgery, Juntendo University School of Medicine, Tokyo 113-8421 , Japan

**Keywords:** aortic valve, annular plication technique, sutureless valve, minimally invasive cardiac surgery

## Abstract

We present the case of a 72-year-old man with symptomatic severe aortic regurgitation and moderate aortic stenosis, and an annular diameter of 30 mm, exceeding the recommended range for sutureless Perceval valve implantation. To reduce the annular size to allow implantation of the Perceval XL valve, an annular plication technique was employed, involving three horizontal mattress sutures placed at the interleaflet triangles. A totally endoscopic approach via right mini-thoracotomy was utilized. Postoperative echocardiography confirmed excellent valve positioning and no paravalvular leak. The patient recovered uneventfully and remained asymptomatic with good prosthesis function at the 3-year follow-up. This case demonstrated that annular plication enables the safe use of the Perceval valve in patients with a mildly oversized annulus and may expand its applicability in minimally invasive settings.

## INTRODUCTION

The Perceval valve (Corcym, Saluggia, Italy), known for its sutureless design, has been widely used in minimally invasive cardiac surgery due to reduced aortic cross-clamp time. However, its use is limited to annular diameters of ≤27 mm. This report describes the annular plication technique for Perceval valve implantation in patients with a mildly oversized annulus.

## SURGICAL TECHNIQUE (VIDEO 1)

A 72-year-old man with symptomatic severe aortic regurgitation and moderate aortic stenosis was considered a candidate for surgical aortic valve replacement. At that time, an automated suture fastening device was not available in the country; therefore, to facilitate implantation via a totally endoscopic approach, the Perceval sutureless valve, offering superior implantability compared with a conventional stented valve, was preferred. Preoperative cardiac computed tomography (CT) revealed a right-noncoronary fused type 1 bicuspid aortic valve with mild annular dilatation (diameter, 30 mm; circumference, 98.6 mm), without significant dilatation of the ascending aorta (32.1 mm) or the sinus of Valsalva (37.6 × 36.0 × 41.0 mm). The largest Perceval valve (XL) is designed for annular diameters ranging from 25 to 27 mm. To achieve a median annular diameter of 26 mm, the circumference needed to be reduced to 81.6 mm. Given the patient’s body size (body surface area was 1.8 m^2^), we anticipated that sufficient effective orifice area (EOA) could be maintained even after annular plication. Therefore, to enable implantation of the Perceval valve, we planned to perform annular plication to reduce the annular size appropriately.

**Figure 1.**
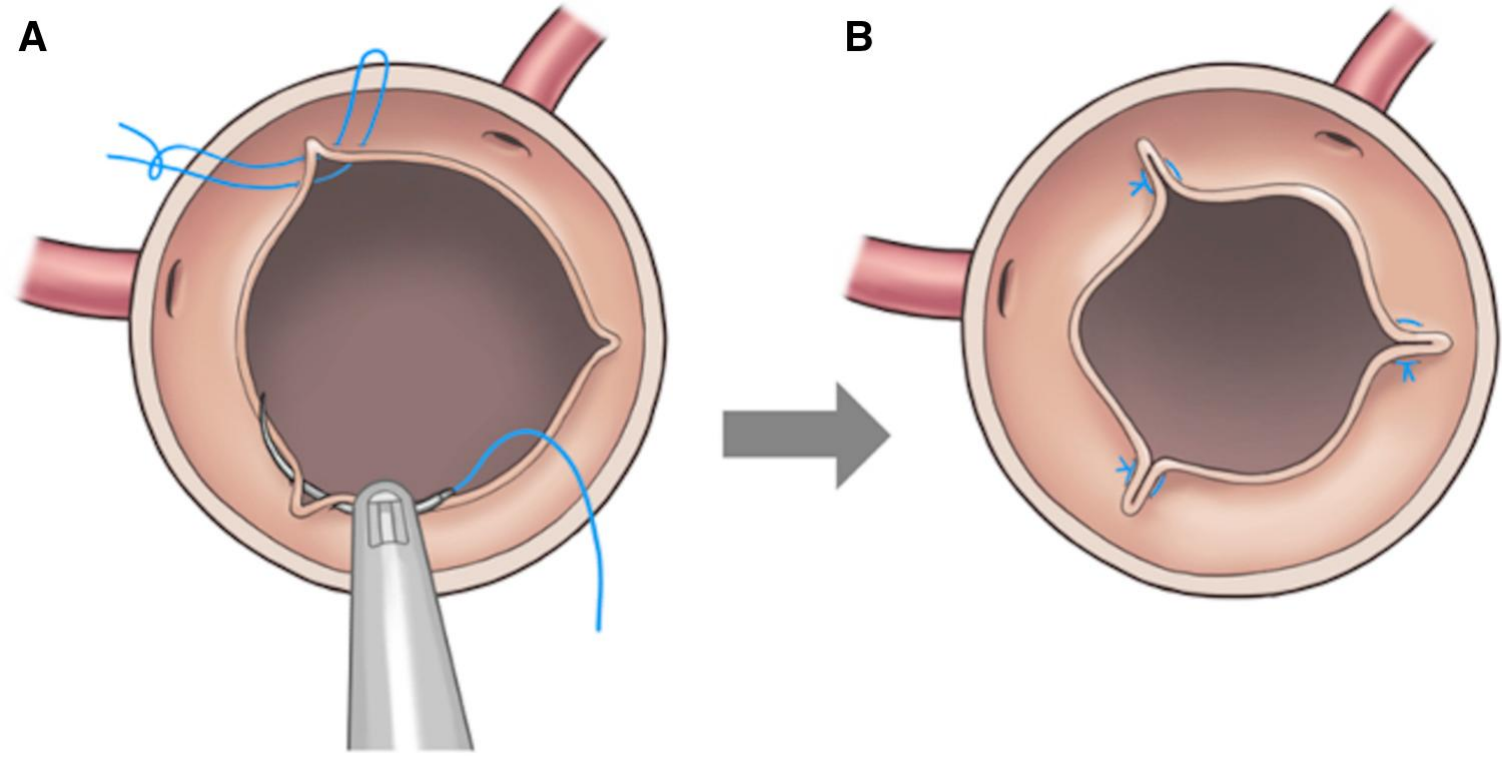
Annular Plication for an Oversized Annulus. (A) U-Shaped Horizontal Mattress Sutures Placed at All Three Interleaflet Triangles. (B) The Annulus Reduced to the Appropriate Size.

**Figure 2.**
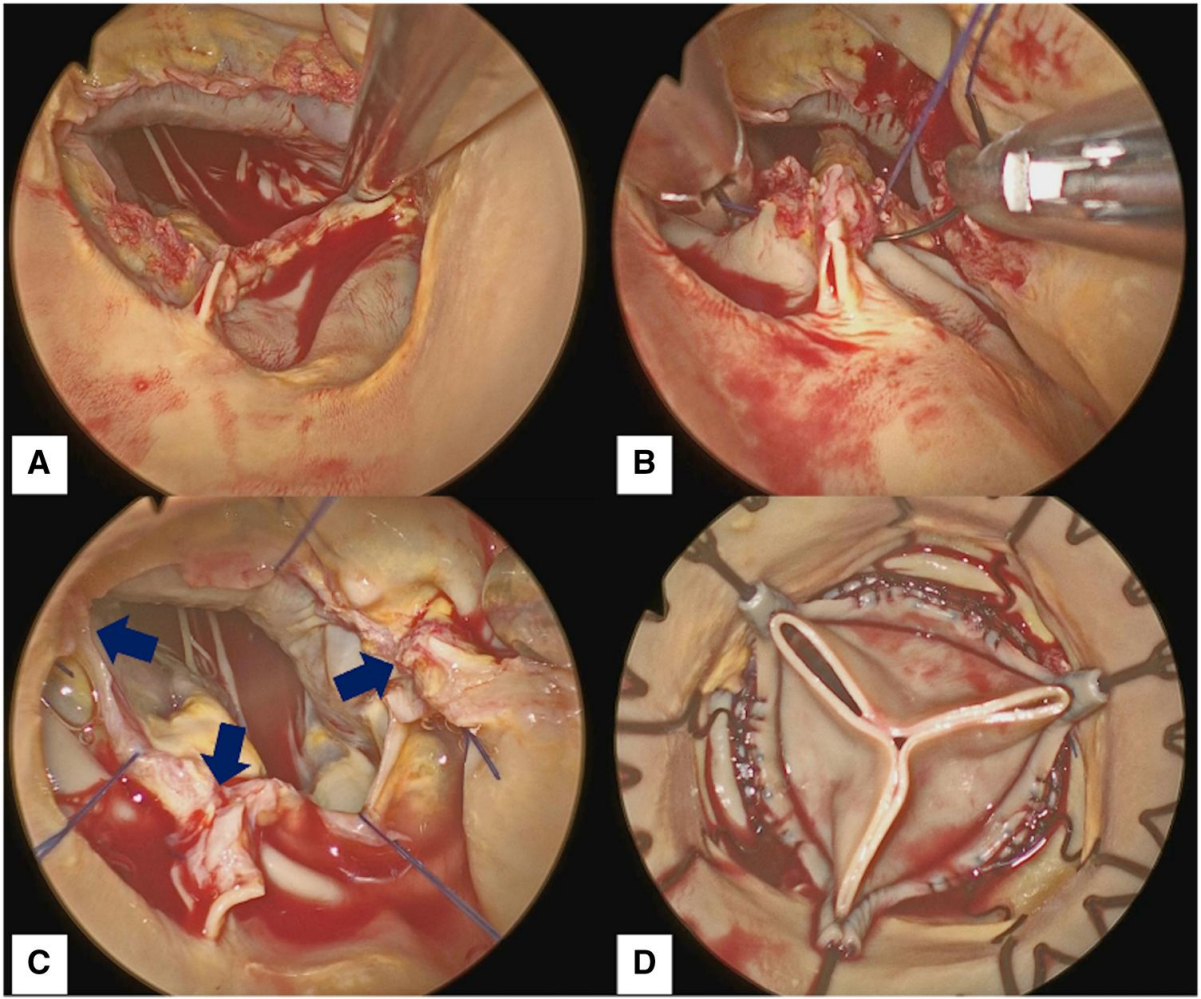
Procedural Steps of Sutureless Valve Implantation with Annular Plication. (A) Decalcified Annulus. (B) A U-Shaped Mattress Suture Placed to Plicate the Interleaflet Triangle. (C) Annular Size Reduced by Plicating All Three Interleaflet Triangles (Arrows). (D) Perceval Valve Implanted in the Plicated Annulus.

The procedure was performed using a totally endoscopic approach through a 3-cm right anterior mini-thoracotomy in the second intercostal space, without a rib spreader, using a 5-mm endoscopic port. A 4 K 30° angled endoscope was employed. Cardiopulmonary bypass (CPB) was initiated via the right femoral artery and vein. After aortic cross-clamping and transverse aortotomy, cardiac asystole was achieved with selective antegrade crystalloid cardioplegia. The calcified cusps were excised, and annular calcification was removed. The raphe height and interleaflet triangle size were comparable to those of the other commissures. Intraoperative sizing confirmed an oversized annulus. Annular plication was performed by reducing approximately 6.0 mm at each interleaflet triangle. Using 4–0 polypropylene sutures, U-shaped horizontal mattress sutures were placed at all 3 interleaflet triangles to achieve annular plication (Figure 1). After plication, sizing confirmed that the annulus had been reduced to the appropriate size for the Perceval XL valve (Figure 2). The prosthesis was then deployed and post-dilated at 4 atmospheres for 30 seconds in a standard manner. CPB and cross-clamp times were 97 and 68 min, respectively. Intraoperative transoesophageal echocardiography confirmed proper valve positioning and the absence of paravalvular leak (PVL).

Postoperative echocardiography revealed excellent prosthesis function with a mean pressure gradient of 8.8 mmHg. The patient was discharged without complications. At the 3-year follow-up, the patient was asymptomatic, and echocardiography showed excellent prosthetic function, with a mean pressure gradient of 6.6 mmHg and no aortic regurgitation. Throughout the postoperative course, no atrioventricular block or dilatation of the ascending aorta or sinus of Valsalva was observed.

## DISCUSSION

The Perceval valve relies on radial force for fixation and is generally recommended for annular sizes of 19-27 mm. In the present case, annular plication enabled implantation despite slightly exceeding this range. During the 3-year follow-up, no PVL or valve migration occurred, demonstrating good mid-term efficacy.

There are 3 key points in the annular plication technique. The first is to estimate the required plication distance based on preoperative CT measurements. The second is to plicate all 3 interleaflet triangles following complete annular decalcification to achieve a balanced annular size reduction. The third is to confirm that the annulus has been reduced to the recommended size post-plication using a sizer.

Potential complications after Perceval implantation with annular plication include PVL^1^ and migration,^2^^,^^3^ especially in cases of incomplete decalcification, undersizing, or bicuspid anatomy. Adherence to the 3 key technical points described above ensured an accurate annular diameter without PVL or migration in the present case. However, this technique is likely unsuitable for patients with connective tissue disorders, such as Marfan syndrome, due to the potential for progressive annular dilatation. Although annular plication inevitably reduces the annular diameter and may raise concerns about a smaller EOA, the Perceval XL valve generally provides sufficient EOA for most patients.^4^ Moreover, because this technique does not reduce coronary height or the diameter of the sinus of Valsalva, it is unlikely to adversely affect the feasibility of future valve-in-valve procedures.

## CONCLUSION

The annular plication technique enabled successful sutureless valve implantation in a patient with a mildly oversized annulus without significant complications during a 3-year follow-up period. This technique expands the applicability of the sutureless valve to a broader patient population.

## Supplementary Material

ivaf287_Supplementary_Data

## Data Availability

The data underlying this article will be shared on reasonable request to the corresponding author.
